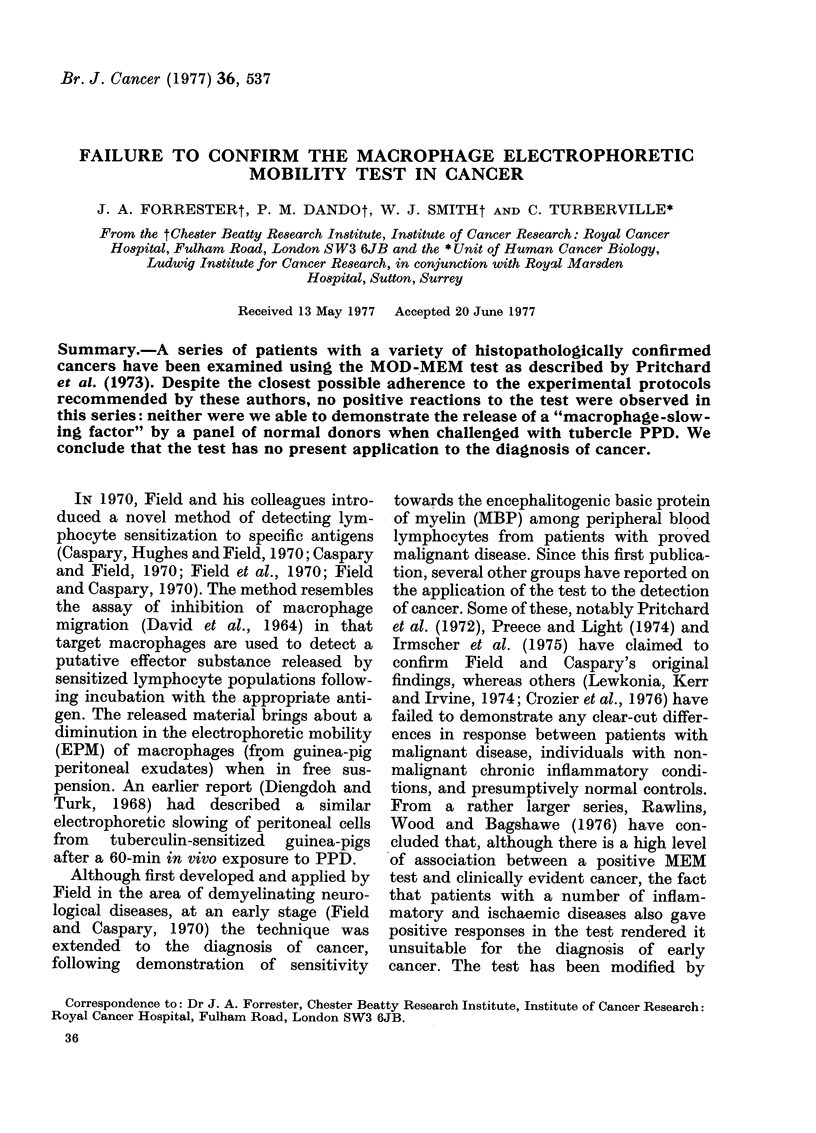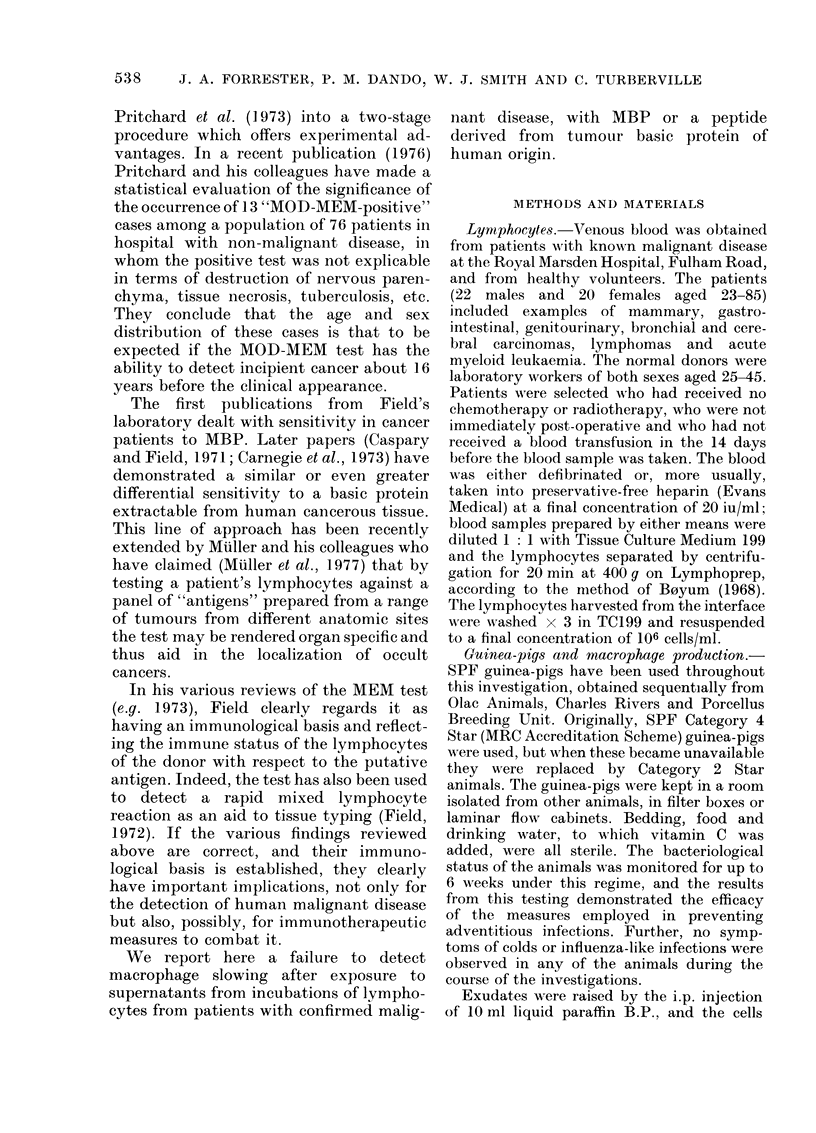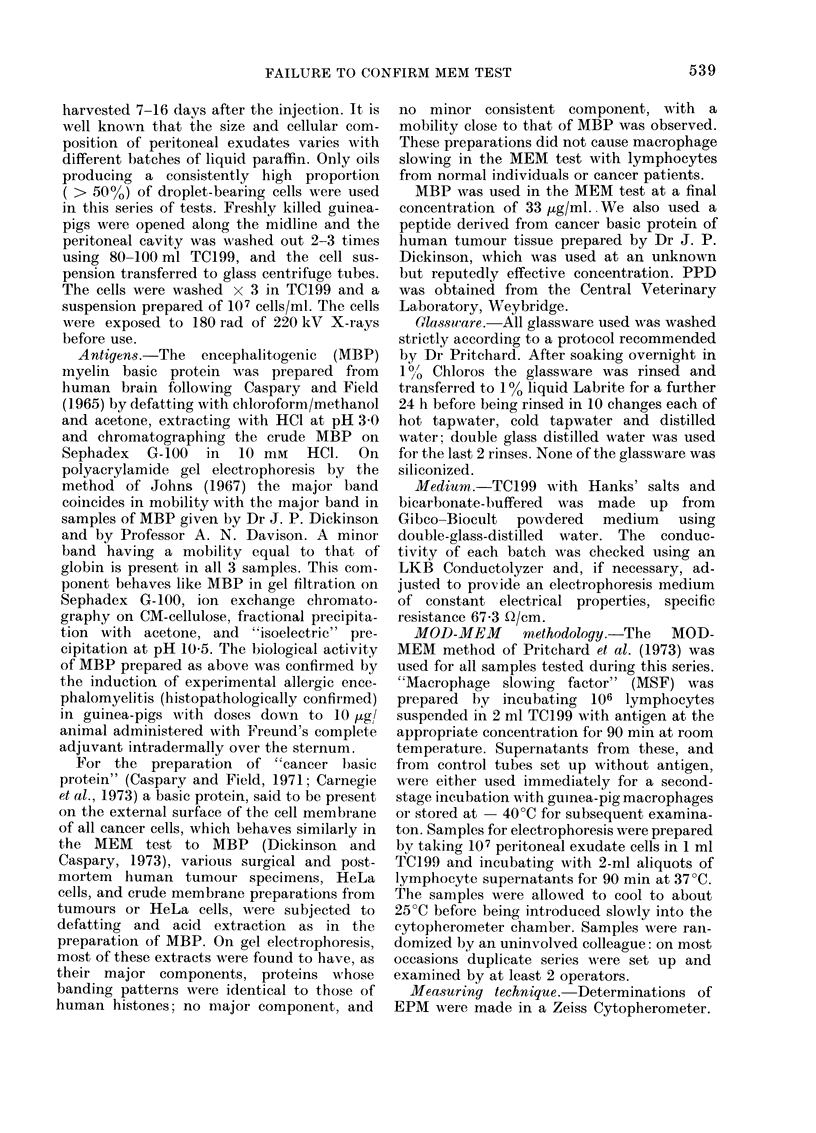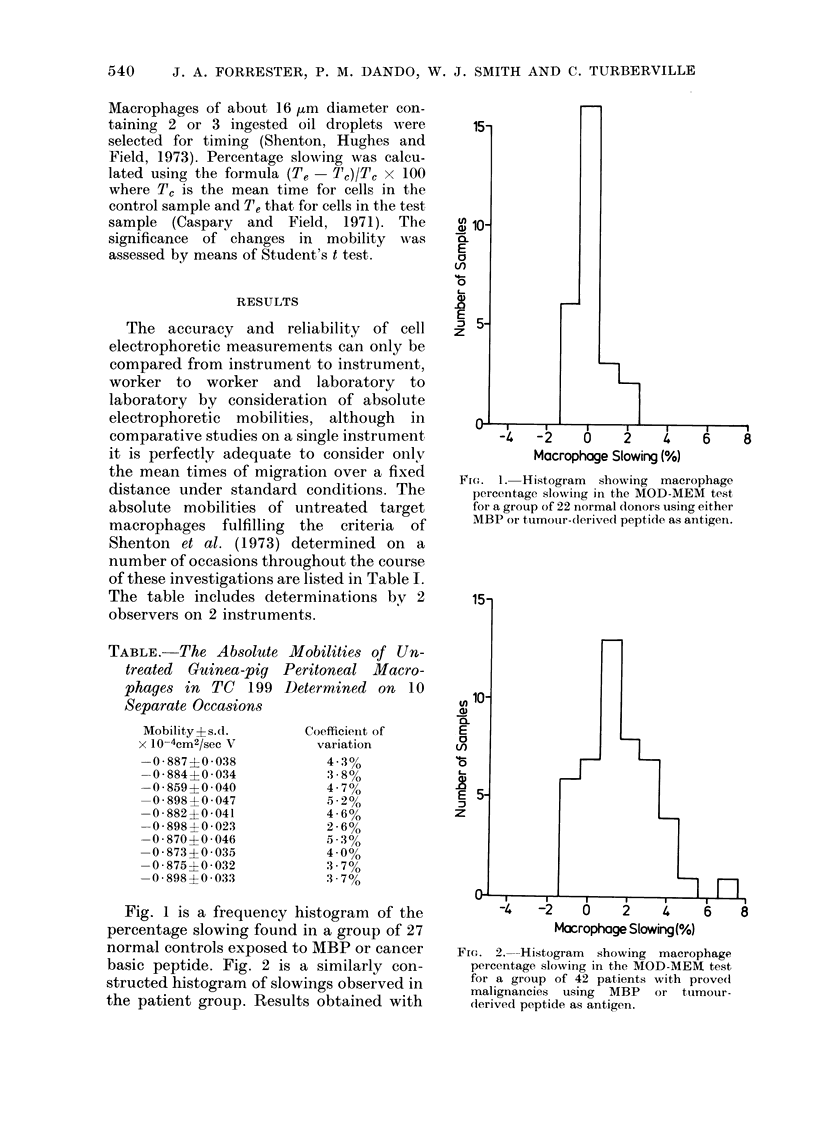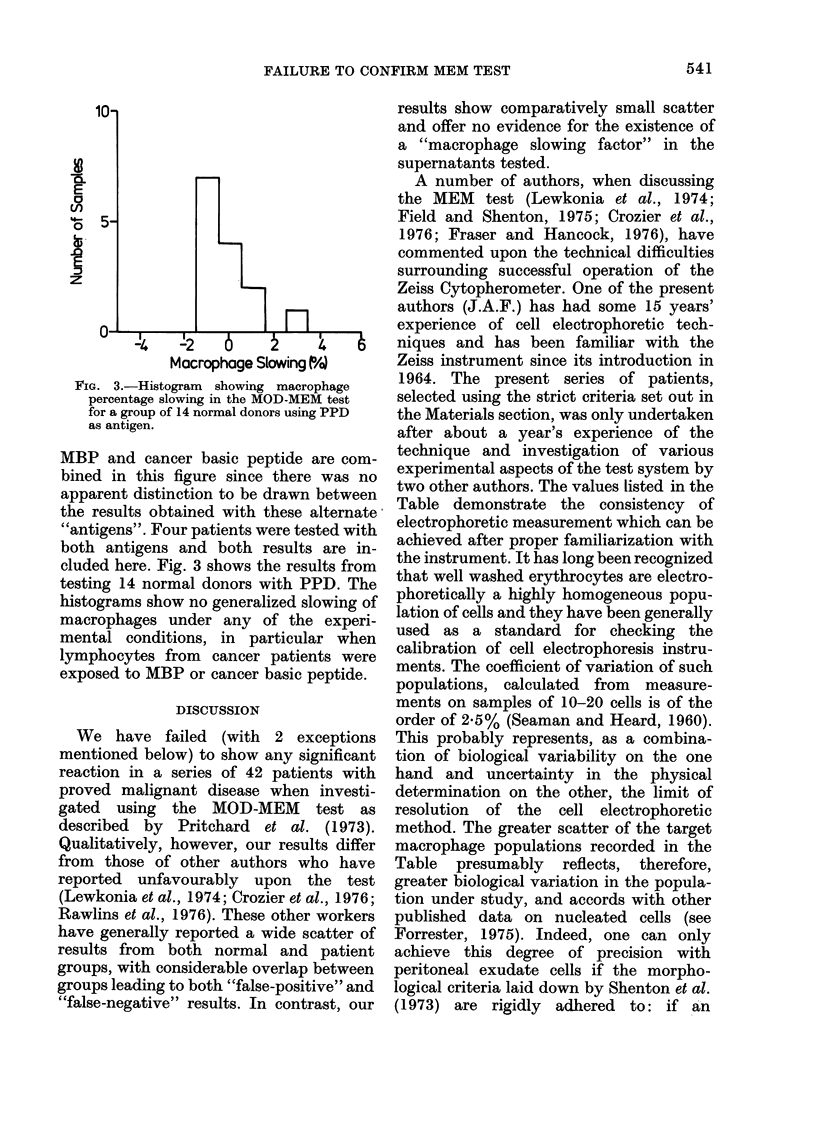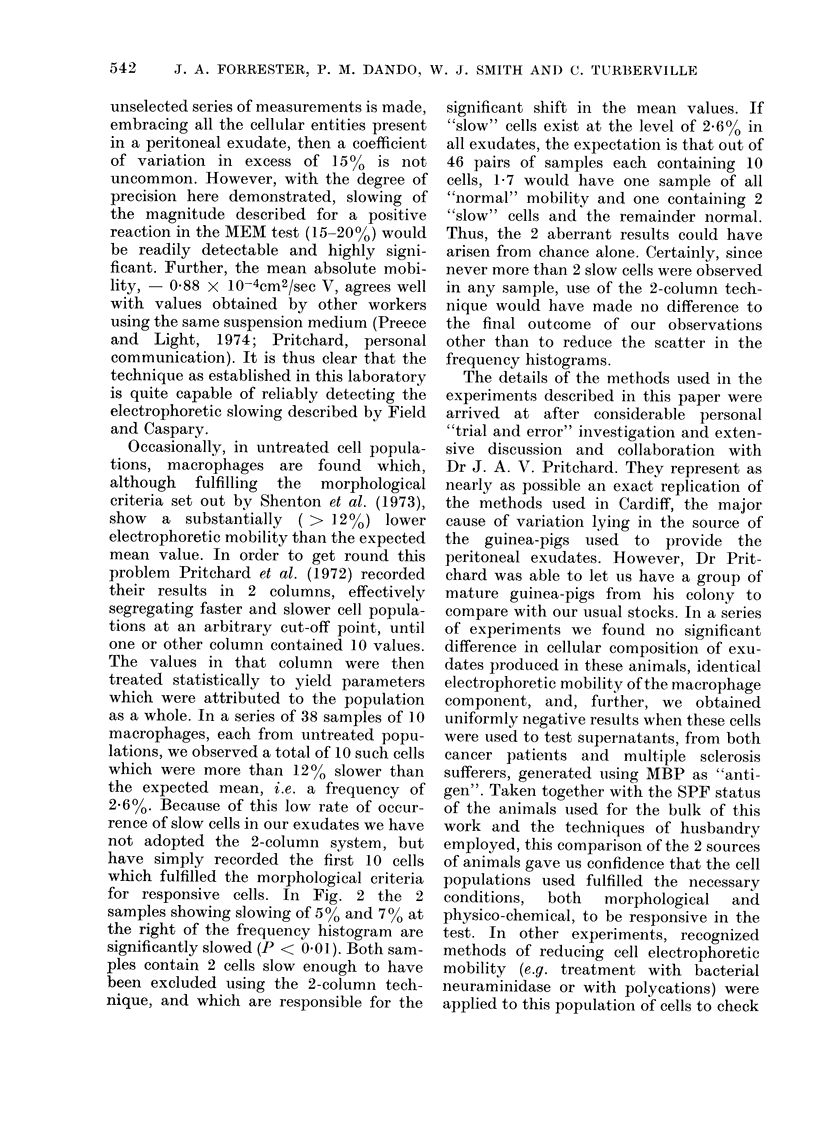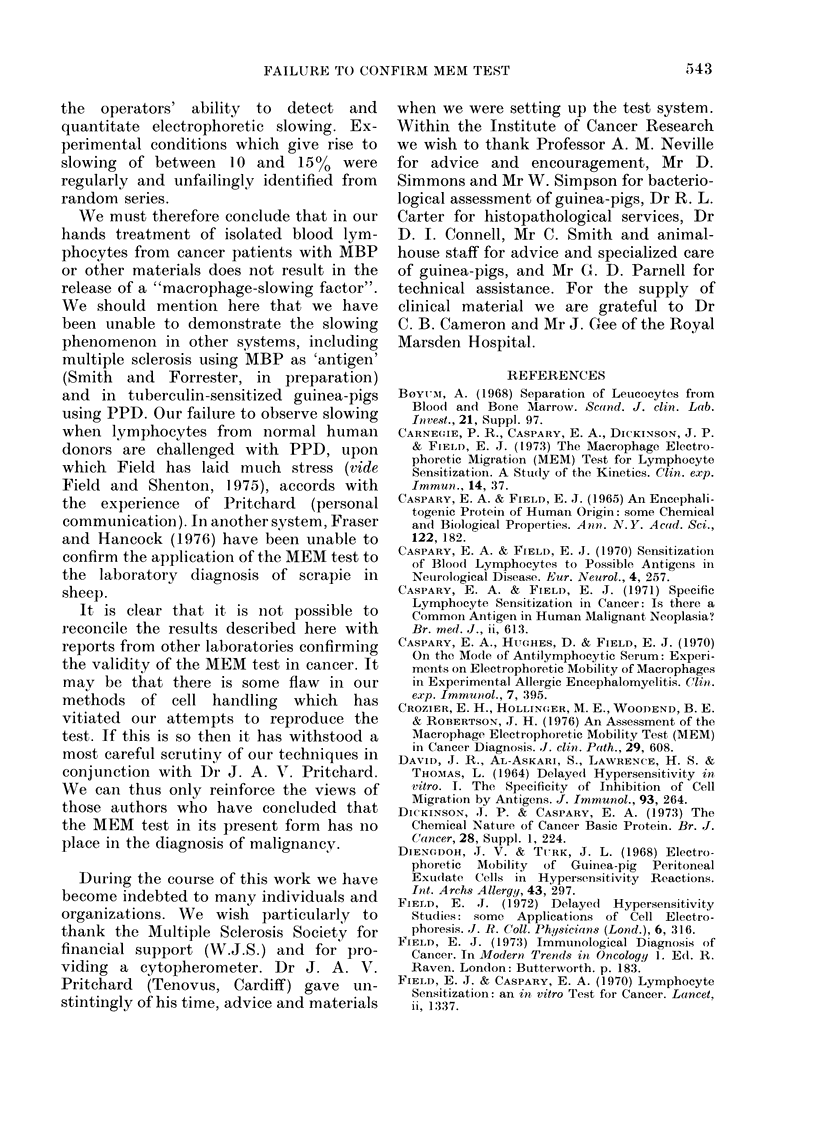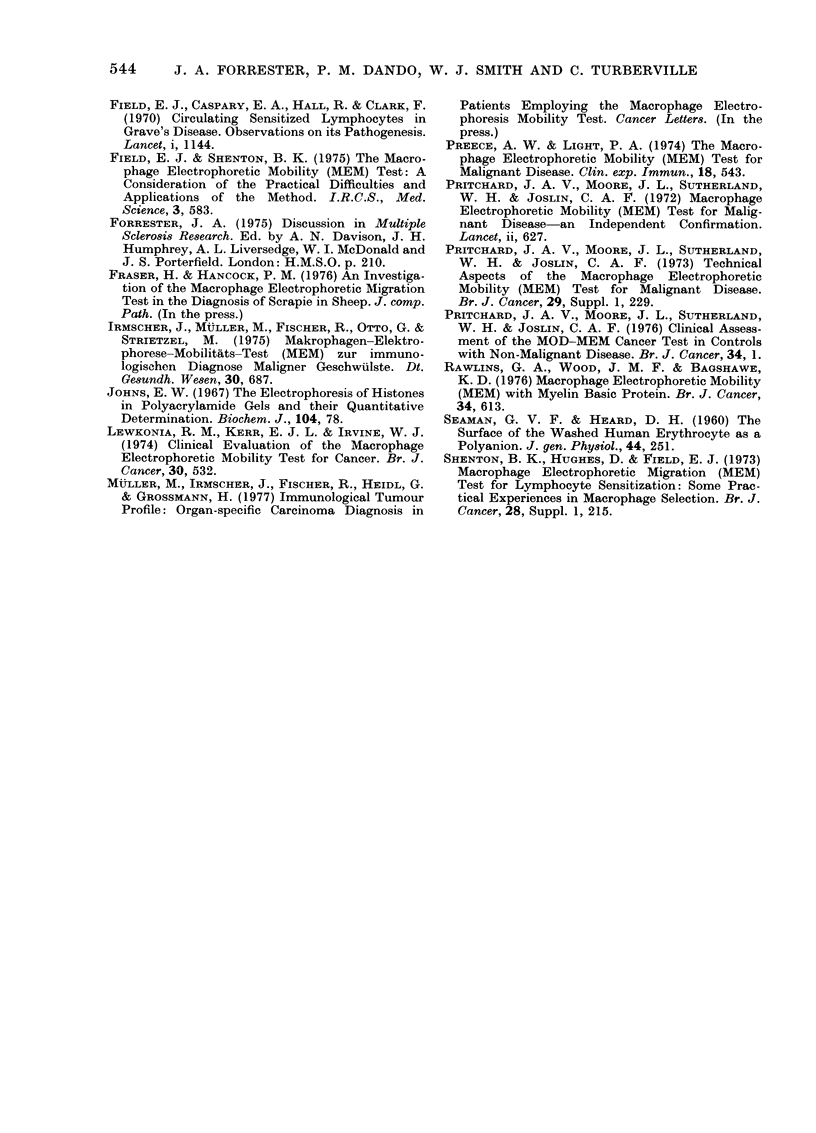# Failure to Confirm the Macrophage Electrophoretic Mobility Test in Cancer

**Published:** 1977-11

**Authors:** J. A. Forrester, P. M. Dando, W. J. Smith, C. Turberville

## Abstract

A series of patients with a variety of histopathologically confirmed cancers have been examined using the MOD-MEM test as described by Pritchard *et al.* (1973). Despite the closest possible adherence to the experimental protocols recommended by these authors, no positive reactions to the test were observed in this series: neither were we able to demonstrate the release of a “macrophage-slowing factor” by a panel of normal donors when challenged with tubercle PPD. We conclude that the test has no present application to the diagnosis of cancer.


*Br. J. Cancer* (1977) **36**, 537

### FAILURE TO CONFIRM THE MACROPHAGE ELECTROPHORETIC MOBILITY TEST IN CANCER

J. A. FORRESTER†, P. M. DANDO†, W. J. SMITH† AND C. TURBERVILLE*

*From the †Chester Beatty Research Institute, Institute of Cancer Research: Royal Cancer Hospital, Fulham Road, London SW3 6JB and the *Unit of Human Cancer Biology, Ludwig Institute for Cancer Research, in conjunction with Royal Marsden Hospital, Sutton, Surrey*

Received 13 May 1977 Accepted 20 June 1977

**Summary.—A series of patients with a variety of histopathologically confirmed cancers have been examined using the MOD-MEM test as described by Pritchard *et al.* (1973). Despite the closest possible adherence to the experimental protocols recommended by these authors, no positive reactions to the test were observed in this series: neither were we able to demonstrate the release of a "macrophage-slowing factor" by a panel of normal donors when challenged with tubercle PPD. We conclude that the test has no present application to the diagnosis of cancer.**

In 1970, Field and his colleagues introduced a novel method of detecting lymphocyte sensitization to specific antigens (Caspary, Hughes and Field, 1970; Caspary and Field, 1970; Field *et al.*, 1970; Field and Caspary, 1970). The method resembles the assay of inhibition of macrophage migration (David *et al.*, 1964) in that target macrophages are used to detect a putative effector substance released by sensitized lymphocyte populations following incubation with the appropriate antigen. The released material brings about a diminution in the electrophoretic mobility (EPM) of macrophages (from guinea-pig peritoneal exudates) when in free suspension. An earlier report (Diengdoh and Turk, 1968) had described a similar electrophoretic slowing of peritoneal cells from tuberculin-sensitized guinea-pigs after a 60-min *in vivo* exposure to PPD.

Although first developed and applied by Field in the area of demyelinating neurological diseases, at an early stage (Field and Caspary, 1970) the technique was extended to the diagnosis of cancer, following demonstration of sensitivity towards the encephalitogenic basic protein of myelin (MBP) among peripheral blood lymphocytes from patients with proved malignant disease. Since this first publication, several other groups have reported on the application of the test to the detection of cancer. Some of these, notably Pritchard *et al.* (1972), Preece and Light (1974) and Irmscher *et al.* (1975) have claimed to confirm Field and Caspary's original findings, whereas others (Lewkonia, Kerr and Irvine, 1974; Crozier *et al.*, 1976) have failed to demonstrate any clear-cut differences in response between patients with malignant disease, individuals with non-malignant chronic inflammatory conditions, and presumptively normal controls. From a rather larger series, Rawlins, Wood and Bagshawe (1976) have concluded that, although there is a high level of association between a positive MEM test and clinically evident cancer, the fact that patients with a number of inflammatory and ischaemic diseases also gave positive responses in the test rendered it unsuitable for the diagnosis of early cancer. The test has been modified by

Correspondence to: Dr J. A. Forrester, Chester Beatty Research Institute, Institute of Cancer Research: Royal Cancer Hospital, Fulham Road, London SW3 6JB.

Pritchard *et al.* (1973) into a two-stage procedure which offers experimental advantages. In a recent publication (1976) Pritchard and his colleagues have made a statistical evaluation of the significance of the occurrence of 13 "MOD-MEM-positive" cases among a population of 76 patients in hospital with non-malignant disease, in whom the positive test was not explicable in terms of destruction of nervous parenchyma, tissue necrosis, tuberculosis, etc. They conclude that the age and sex distribution of these cases is that to be expected if the MOD-MEM test has the ability to detect incipient cancer about 16 years before the clinical appearance.

The first publications from Field's laboratory dealt with sensitivity in cancer patients to MBP. Later papers (Caspary and Field, 1971; Carnegie *et al.*, 1973) have demonstrated a similar or even greater differential sensitivity to a basic protein extractable from human cancerous tissue. This line of approach has been recently extended by Müller and his colleagues who have claimed (Müller *et al.*, 1977) that by testing a patient's lymphocytes against a panel of "antigens" prepared from a range of tumours from different anatomic sites the test may be rendered organ specific and thus aid in the localization of occult cancers.

In his various reviews of the MEM test (*e.g.* 1973), Field clearly regards it as having an immunological basis and reflecting the immune status of the lymphocytes of the donor with respect to the putative antigen. Indeed, the test has also been used to detect a rapid mixed lymphocyte reaction as an aid to tissue typing (Field, 1972). If the various findings reviewed above are correct, and their immunological basis is established, they clearly have important implications, not only for the detection of human malignant disease but also, possibly, for immunotherapeutic measures to combat it.

We report here a failure to detect macrophage slowing after exposure to supernatants from incubations of lymphocytes from patients with confirmed malignant disease, with MBP or a peptide derived from tumour basic protein of human origin.

### METHODS AND MATERIALS

*Lymphocytes.*—Venous blood was obtained from patients with known malignant disease at the Royal Marsden Hospital, Fulham Road, and from healthy volunteers. The patients (22 males and 20 females aged 23–85) included examples of mammary, gastrointestinal, genitourinary, bronchial and cerebral carcinomas, lymphomas and acute myeloid leukaemia. The normal donors were laboratory workers of both sexes aged 25–45. Patients were selected who had received no chemotherapy or radiotherapy, who were not immediately post-operative and who had not received a blood transfusion in the 14 days before the blood sample was taken. The blood was either defibrinated or, more usually, taken into preservative-free heparin (Evans Medical) at a final concentration of 20 iu/ml; blood samples prepared by either means were diluted 1 : 1 with Tissue Culture Medium 199 and the lymphocytes separated by centrifugation for 20 min at 400 *g* on Lymphoprep, according to the method of Bøyum (1968). The lymphocytes harvested from the interface were washed $\times$ 3 in TC199 and resuspended to a final concentration of $10^6$ cells/ml.

*Guinea-pigs and macrophage production.*—SPF guinea-pigs have been used throughout this investigation, obtained sequentially from Olac Animals, Charles Rivers and Porcellus Breeding Unit. Originally, SPF Category 4 Star (MRC Accreditation Scheme) guinea-pigs were used, but when these became unavailable they were replaced by Category 2 Star animals. The guinea-pigs were kept in a room isolated from other animals, in filter boxes or laminar flow cabinets. Bedding, food and drinking water, to which vitamin C was added, were all sterile. The bacteriological status of the animals was monitored for up to 6 weeks under this regime, and the results from this testing demonstrated the efficacy of the measures employed in preventing adventitious infections. Further, no symptoms of colds or influenza-like infections were observed in any of the animals during the course of the investigations.

Exudates were raised by the i.p. injection of 10 ml liquid paraffin B.P., and the cells harvested 7–16 days after the injection. It is well known that the size and cellular composition of peritoneal exudates varies with different batches of liquid paraffin. Only oils producing a consistently high proportion ($>50\%$) of droplet-bearing cells were used in this series of tests. Freshly killed guinea-pigs were opened along the midline and the peritoneal cavity was washed out 2–3 times using 80–100 ml TC199, and the cell suspension transferred to glass centrifuge tubes. The cells were washed $\times 3$ in TC199 and a suspension prepared of $10^7$ cells/ml. The cells were exposed to 180 rad of 220 kV X-rays before use.

*Antigens.*—The encephalitogenic (MBP) myelin basic protein was prepared from human brain following Caspary and Field (1965) by defatting with chloroform/methanol and acetone, extracting with HCl at pH 3·0 and chromatographing the crude MBP on Sephadex G-100 in 10 mM HCl. On polyacrylamide gel electrophoresis by the method of Johns (1967) the major band coincides in mobility with the major band in samples of MBP given by Dr J. P. Dickinson and by Professor A. N. Davison. A minor band having a mobility equal to that of globin is present in all 3 samples. This component behaves like MBP in gel filtration on Sephadex G-100, ion exchange chromatography on CM-cellulose, fractional precipitation with acetone, and "isoelectric" precipitation at pH 10·5. The biological activity of MBP prepared as above was confirmed by the induction of experimental allergic encephalomyelitis (histopathologically confirmed) in guinea-pigs with doses down to 10 $\mu$g/animal administered with Freund's complete adjuvant intradermally over the sternum.

For the preparation of "cancer basic protein" (Caspary and Field, 1971; Carnegie *et al.*, 1973) a basic protein, said to be present on the external surface of the cell membrane of all cancer cells, which behaves similarly in the MEM test to MBP (Dickinson and Caspary, 1973), various surgical and post-mortem human tumour specimens, HeLa cells, and crude membrane preparations from tumours or HeLa cells, were subjected to defatting and acid extraction as in the preparation of MBP. On gel electrophoresis, most of these extracts were found to have, as their major components, proteins whose banding patterns were identical to those of human histones; no major component, and no minor consistent component, with a mobility close to that of MBP was observed. These preparations did not cause macrophage slowing in the MEM test with lymphocytes from normal individuals or cancer patients.

MBP was used in the MEM test at a final concentration of 33 $\mu$g/ml. We also used a peptide derived from cancer basic protein of human tumour tissue prepared by Dr J. P. Dickinson, which was used at an unknown but reputedly effective concentration. PPD was obtained from the Central Veterinary Laboratory, Weybridge.

*Glassware.*—All glassware used was washed strictly according to a protocol recommended by Dr Pritchard. After soaking overnight in 1% Chloros the glassware was rinsed and transferred to 1% liquid Labrite for a further 24 h before being rinsed in 10 changes each of hot tapwater, cold tapwater and distilled water; double glass distilled water was used for the last 2 rinses. None of the glassware was siliconized.

*Medium.*—TC199 with Hanks' salts and bicarbonate-buffered was made up from Gibco–Biocult powdered medium using double-glass-distilled water. The conductivity of each batch was checked using an LKB Conductolyzer and, if necessary, adjusted to provide an electrophoresis medium of constant electrical properties, specific resistance 67·3 Ω/cm.

*MOD-MEM methodology.*—The MOD-MEM method of Pritchard *et al.* (1973) was used for all samples tested during this series. "Macrophage slowing factor" (MSF) was prepared by incubating $10^6$ lymphocytes suspended in 2 ml TC199 with antigen at the appropriate concentration for 90 min at room temperature. Supernatants from these, and from control tubes set up without antigen, were either used immediately for a second-stage incubation with guinea-pig macrophages or stored at $-40$°C for subsequent examinaton. Samples for electrophoresis were prepared by taking $10^7$ peritoneal exudate cells in 1 ml TC199 and incubating with 2-ml aliquots of lymphocyte supernatants for 90 min at 37°C. The samples were allowed to cool to about 25°C before being introduced slowly into the cytopherometer chamber. Samples were randomized by an uninvolved colleague: on most occasions duplicate series were set up and examined by at least 2 operators.

*Measuring technique.*—Determinations of EPM were made in a Zeiss Cytopherometer.

Macrophages of about 16 $\mu$m diameter containing 2 or 3 ingested oil droplets were selected for timing (Shenton, Hughes and Field, 1973). Percentage slowing was calculated using the formula $(T_e - T_c)/T_c \times 100$ where $T_c$ is the mean time for cells in the control sample and $T_e$ that for cells in the test sample (Caspary and Field, 1971). The significance of changes in mobility was assessed by means of Student's $t$ test.

### RESULTS

The accuracy and reliability of cell electrophoretic measurements can only be compared from instrument to instrument, worker to worker and laboratory to laboratory by consideration of absolute electrophoretic mobilities, although in comparative studies on a single instrument it is perfectly adequate to consider only the mean times of migration over a fixed distance under standard conditions. The absolute mobilities of untreated target macrophages fulfilling the criteria of Shenton *et al.* (1973) determined on a number of occasions throughout the course of these investigations are listed in Table I. The table includes determinations by 2 observers on 2 instruments.

TABLE.—*The Absolute Mobilities of Untreated Guinea-pig Peritoneal Macrophages in TC* 199 *Determined on* 10 *Separate Occasions*

| Mobility ± s.d. $\times 10^{-4}$cm$^2$/sec V | Coefficient of variation |
|---|---|
| $-0 \cdot 887 \pm 0 \cdot 038$ | 4·3% |
| $-0 \cdot 884 \pm 0 \cdot 034$ | 3·8% |
| $-0 \cdot 859 \pm 0 \cdot 040$ | 4·7% |
| $-0 \cdot 898 \pm 0 \cdot 047$ | 5·2% |
| $-0 \cdot 882 \pm 0 \cdot 041$ | 4·6% |
| $-0 \cdot 898 \pm 0 \cdot 023$ | 2·6% |
| $-0 \cdot 870 \pm 0 \cdot 046$ | 5·3% |
| $-0 \cdot 873 \pm 0 \cdot 035$ | 4·0% |
| $-0 \cdot 875 \pm 0 \cdot 032$ | 3·7% |
| $-0 \cdot 898 \pm 0 \cdot 033$ | 3·7% |

Fig. 1 is a frequency histogram of the percentage slowing found in a group of 27 normal controls exposed to MBP or cancer basic peptide. Fig. 2 is a similarly constructed histogram of slowings observed in the patient group. Results obtained with

FIG. 1.—Histogram showing macrophage percentage slowing in the MOD-MEM test for a group of 22 normal donors using either MBP or tumour-derived peptide as antigen.

FIG. 2.—Histogram showing macrophage percentage slowing in the MOD-MEM test for a group of 42 patients with proved malignancies using MBP or tumour-derived peptide as antigen.

FIG. 3.—Histogram showing macrophage percentage slowing in the MOD-MEM test for a group of 14 normal donors using PPD as antigen.

MBP and cancer basic peptide are combined in this figure since there was no apparent distinction to be drawn between the results obtained with these alternate "antigens". Four patients were tested with both antigens and both results are included here. Fig. 3 shows the results from testing 14 normal donors with PPD. The histograms show no generalized slowing of macrophages under any of the experimental conditions, in particular when lymphocytes from cancer patients were exposed to MBP or cancer basic peptide.

### DISCUSSION

We have failed (with 2 exceptions mentioned below) to show any significant reaction in a series of 42 patients with proved malignant disease when investigated using the MOD-MEM test as described by Pritchard *et al.* (1973). Qualitatively, however, our results differ from those of other authors who have reported unfavourably upon the test (Lewkonia *et al.*, 1974; Crozier *et al.*, 1976; Rawlins *et al.*, 1976). These other workers have generally reported a wide scatter of results from both normal and patient groups, with considerable overlap between groups leading to both "false-positive" and "false-negative" results. In contrast, our results show comparatively small scatter and offer no evidence for the existence of a "macrophage slowing factor" in the supernatants tested.

A number of authors, when discussing the MEM test (Lewkonia *et al.*, 1974; Field and Shenton, 1975; Crozier *et al.*, 1976; Fraser and Hancock, 1976), have commented upon the technical difficulties surrounding successful operation of the Zeiss Cytopherometer. One of the present authors (J.A.F.) has had some 15 years' experience of cell electrophoretic techniques and has been familiar with the Zeiss instrument since its introduction in 1964. The present series of patients, selected using the strict criteria set out in the Materials section, was only undertaken after about a year's experience of the technique and investigation of various experimental aspects of the test system by two other authors. The values listed in the Table demonstrate the consistency of electrophoretic measurement which can be achieved after proper familiarization with the instrument. It has long been recognized that well washed erythrocytes are electrophoretically a highly homogeneous population of cells and they have been generally used as a standard for checking the calibration of cell electrophoresis instruments. The coefficient of variation of such populations, calculated from measurements on samples of 10–20 cells is of the order of 2·5% (Seaman and Heard, 1960). This probably represents, as a combination of biological variability on the one hand and uncertainty in the physical determination on the other, the limit of resolution of the cell electrophoretic method. The greater scatter of the target macrophage populations recorded in the Table presumably reflects, therefore, greater biological variation in the population under study, and accords with other published data on nucleated cells (see Forrester, 1975). Indeed, one can only achieve this degree of precision with peritoneal exudate cells if the morphological criteria laid down by Shenton *et al.* (1973) are rigidly adhered to: if an unselected series of measurements is made, embracing all the cellular entities present in a peritoneal exudate, then a coefficient of variation in excess of 15% is not uncommon. However, with the degree of precision here demonstrated, slowing of the magnitude described for a positive reaction in the MEM test (15–20%) would be readily detectable and highly significant. Further, the mean absolute mobility, $- 0{\cdot}88 \times 10^{-4} cm^2/sec\ V$, agrees well with values obtained by other workers using the same suspension medium (Preece and Light, 1974; Pritchard, personal communication). It is thus clear that the technique as established in this laboratory is quite capable of reliably detecting the electrophoretic slowing described by Field and Caspary.

Occasionally, in untreated cell populations, macrophages are found which, although fulfilling the morphological criteria set out by Shenton *et al.* (1973), show a substantially ($> 12\%$) lower electrophoretic mobility than the expected mean value. In order to get round this problem Pritchard *et al.* (1972) recorded their results in 2 columns, effectively segregating faster and slower cell populations at an arbitrary cut-off point, until one or other column contained 10 values. The values in that column were then treated statistically to yield parameters which were attributed to the population as a whole. In a series of 38 samples of 10 macrophages, each from untreated populations, we observed a total of 10 such cells which were more than 12% slower than the expected mean, *i.e.* a frequency of 2·6%. Because of this low rate of occurrence of slow cells in our exudates we have not adopted the 2-column system, but have simply recorded the first 10 cells which fulfilled the morphological criteria for responsive cells. In Fig. 2 the 2 samples showing slowing of 5% and 7% at the right of the frequency histogram are significantly slowed ($P < 0{\cdot}01$). Both samples contain 2 cells slow enough to have been excluded using the 2-column technique, and which are responsible for the significant shift in the mean values. If "slow" cells exist at the level of 2·6% in all exudates, the expectation is that out of 46 pairs of samples each containing 10 cells, 1·7 would have one sample of all "normal" mobility and one containing 2 "slow" cells and the remainder normal. Thus, the 2 aberrant results could have arisen from chance alone. Certainly, since never more than 2 slow cells were observed in any sample, use of the 2-column technique would have made no difference to the final outcome of our observations other than to reduce the scatter in the frequency histograms.

The details of the methods used in the experiments described in this paper were arrived at after considerable personal "trial and error" investigation and extensive discussion and collaboration with Dr J. A. V. Pritchard. They represent as nearly as possible an exact replication of the methods used in Cardiff, the major cause of variation lying in the source of the guinea-pigs used to provide the peritoneal exudates. However, Dr Pritchard was able to let us have a group of mature guinea-pigs from his colony to compare with our usual stocks. In a series of experiments we found no significant difference in cellular composition of exudates produced in these animals, identical electrophoretic mobility of the macrophage component, and, further, we obtained uniformly negative results when these cells were used to test supernatants, from both cancer patients and multiple sclerosis sufferers, generated using MBP as "antigen". Taken together with the SPF status of the animals used for the bulk of this work and the techniques of husbandry employed, this comparison of the 2 sources of animals gave us confidence that the cell populations used fulfilled the necessary conditions, both morphological and physico-chemical, to be responsive in the test. In other experiments, recognized methods of reducing cell electrophoretic mobility (*e.g.* treatment with bacterial neuraminidase or with polycations) were applied to this population of cells to check the operators' ability to detect and quantitate electrophoretic slowing. Experimental conditions which give rise to slowing of between 10 and 15% were regularly and unfailingly identified from random series.

We must therefore conclude that in our hands treatment of isolated blood lymphocytes from cancer patients with MBP or other materials does not result in the release of a "macrophage-slowing factor". We should mention here that we have been unable to demonstrate the slowing phenomenon in other systems, including multiple sclerosis using MBP as 'antigen' (Smith and Forrester, in preparation) and in tuberculin-sensitized guinea-pigs using PPD. Our failure to observe slowing when lymphocytes from normal human donors are challenged with PPD, upon which Field has laid much stress (*vide* Field and Shenton, 1975), accords with the experience of Pritchard (personal communication). In another system, Fraser and Hancock (1976) have been unable to confirm the application of the MEM test to the laboratory diagnosis of scrapie in sheep.

It is clear that it is not possible to reconcile the results described here with reports from other laboratories confirming the validity of the MEM test in cancer. It may be that there is some flaw in our methods of cell handling which has vitiated our attempts to reproduce the test. If this is so then it has withstood a most careful scrutiny of our techniques in conjunction with Dr J. A. V. Pritchard. We can thus only reinforce the views of those authors who have concluded that the MEM test in its present form has no place in the diagnosis of malignancy.

During the course of this work we have become indebted to many individuals and organizations. We wish particularly to thank the Multiple Sclerosis Society for financial support (W.J.S.) and for providing a cytopherometer. Dr J. A. V. Pritchard (Tenovus, Cardiff) gave unstintingly of his time, advice and materials when we were setting up the test system. Within the Institute of Cancer Research we wish to thank Professor A. M. Neville for advice and encouragement, Mr D. Simmons and Mr W. Simpson for bacteriological assessment of guinea-pigs, Dr R. L. Carter for histopathological services, Dr D. I. Connell, Mr C. Smith and animal-house staff for advice and specialized care of guinea-pigs, and Mr G. D. Parnell for technical assistance. For the supply of clinical material we are grateful to Dr C. B. Cameron and Mr J. Gee of the Royal Marsden Hospital.

### REFERENCES

Bøyum, A. (1968) Separation of Leucocytes from Blood and Bone Marrow. *Scand. J. clin. Lab. Invest.*, **21**, Suppl. 97.

Carnegie, P. R., Caspary, E. A., Dickinson, J. P. & Field, E. J. (1973) The Macrophage Electrophoretic Migration (MEM) Test for Lymphocyte Sensitization. A Study of the Kinetics. *Clin. exp. Immun.*, **14**, 37.

Caspary, E. A. & Field, E. J. (1965) An Encephalitogenic Protein of Human Origin: some Chemical and Biological Properties. *Ann. N.Y. Acad. Sci.*, **122**, 182.

Caspary, E. A. & Field, E. J. (1970) Sensitization of Blood Lymphocytes to Possible Antigens in Neurological Disease. *Eur. Neurol.*, **4**, 257.

Caspary, E. A. & Field, E. J. (1971) Specific Lymphocyte Sensitization in Cancer: Is there a Common Antigen in Human Malignant Neoplasia? *Br. med. J.*, ii, 613.

Caspary, E. A., Hughes, D. & Field, E. J. (1970) On the Mode of Antilymphocytic Serum: Experiments on Electrophoretic Mobility of Macrophages in Experimental Allergic Encephalomyelitis. *Clin. exp. Immunol.*, **7**, 395.

Crozier, E. H., Hollinger, M. E., Woodend, B. E. & Robertson, J. H. (1976) An Assessment of the Macrophage Electrophoretic Mobility Test (MEM) in Cancer Diagnosis. *J. clin. Path.*, **29**, 608.

David, J. R., Al-Askari, S., Lawrence, H. S. & Thomas, L. (1964) Delayed Hypersensitivity *in vitro*. I. The Specificity of Inhibition of Cell Migration by Antigens. *J. Immunol.*, **93**, 264.

Dickinson, J. P. & Caspary, E. A. (1973) The Chemical Nature of Cancer Basic Protein. *Br. J. Cancer*, **28**, Suppl. 1, 224.

Diengdoh, J. V. & Turk, J. L. (1968) Electrophoretic Mobility of Guinea-pig Peritoneal Exudate Cells in Hypersensitivity Reactions. *Int. Archs Allergy*, **43**, 297.

Field, E. J. (1972) Delayed Hypersensitivity Studies: some Applications of Cell Electrophoresis. *J. R. Coll. Physicians* (*Lond.*), **6**, 316.

Field, E. J. (1973) Immunological Diagnosis of Cancer. In *Modern Trends in Oncology* 1. Ed. R. Raven. London: Butterworth. p. 183.

Field, E. J. & Caspary, E. A. (1970) Lymphocyte Sensitization: an *in vitro* Test for Cancer. *Lancet*, ii, 1337.

FIELD, E. J., CASPARY, E. A., HALL, R. & CLARK, F. (1970) Circulating Sensitized Lymphocytes in Grave's Disease. Observations on its Pathogenesis. *Lancet*, i, 1144.

FIELD, E. J. & SHENTON, B. K. (1975) The Macrophage Electrophoretic Mobility (MEM) Test: A Consideration of the Practical Difficulties and Applications of the Method. *I.R.C.S., Med. Science*, **3**, 583.

FORRESTER, J. A. (1975) Discussion in *Multiple Sclerosis Research*. Ed. by A. N. Davison, J. H. Humphrey, A. L. Liversedge, W. I. McDonald and J. S. Porterfield. London: H.M.S.O. p. 210.

FRASER, H. & HANCOCK, P. M. (1976) An Investigation of the Macrophage Electrophoretic Migration Test in the Diagnosis of Scrapie in Sheep. *J. comp. Path.* (In the press.)

IRMSCHER, J., MÜLLER, M., FISCHER, R., OTTO, G. & STRIETZEL, M. (1975) Makrophagen–Elektrophorese–Mobilitäts–Test (MEM) zur immunologischen Diagnose Maligner Geschwülste. *Dt. Gesundh. Wesen*, **30**, 687.

JOHNS, E. W. (1967) The Electrophoresis of Histones in Polyacrylamide Gels and their Quantitative Determination. *Biochem. J.*, **104**, 78.

LEWKONIA, R. M., KERR, E. J. L. & IRVINE, W. J. (1974) Clinical Evaluation of the Macrophage Electrophoretic Mobility Test for Cancer. *Br. J. Cancer*, **30**, 532.

MÜLLER, M., IRMSCHER, J., FISCHER, R., HEIDL, G. & GROSSMANN, H. (1977) Immunological Tumour Profile: Organ-specific Carcinoma Diagnosis in Patients Employing the Macrophage Electrophoresis Mobility Test. *Cancer Letters*. (In the press.)

PREECE, A. W. & LIGHT, P. A. (1974) The Macrophage Electrophoretic Mobility (MEM) Test for Malignant Disease. *Clin. exp. Immun.*, **18**, 543.

PRITCHARD, J. A. V., MOORE, J. L., SUTHERLAND, W. H. & JOSLIN, C. A. F. (1972) Macrophage Electrophoretic Mobility (MEM) Test for Malignant Disease—an Independent Confirmation. *Lancet*, ii, 627.

PRITCHARD, J. A. V., MOORE, J. L., SUTHERLAND, W. H. & JOSLIN, C. A. F. (1973) Technical Aspects of the Macrophage Electrophoretic Mobility (MEM) Test for Malignant Disease. *Br. J. Cancer*, **29**, Suppl. 1, 229.

PRITCHARD, J. A. V., MOORE, J. L., SUTHERLAND, W. H. & JOSLIN, C. A. F. (1976) Clinical Assessment of the MOD–MEM Cancer Test in Controls with Non-Malignant Disease. *Br. J. Cancer*, **34**, 1.

RAWLINS, G. A., WOOD, J. M. F. & BAGSHAWE, K. D. (1976) Macrophage Electrophoretic Mobility (MEM) with Myelin Basic Protein. *Br. J. Cancer*, **34**, 613.

SEAMAN, G. V. F. & HEARD, D. H. (1960) The Surface of the Washed Human Erythrocyte as a Polyanion. *J. gen. Physiol.*, **44**, 251.

SHENTON, B. K., HUGHES, D. & FIELD, E. J. (1973) Macrophage Electrophoretic Migration (MEM) Test for Lymphocyte Sensitization: Some Practical Experiences in Macrophage Selection. *Br. J. Cancer*, **28**, Suppl. 1, 215.